# Composition and Surface Optical Properties of GaSe:Eu Crystals before and after Heat Treatment

**DOI:** 10.3390/ma17020405

**Published:** 2024-01-13

**Authors:** Veaceslav Sprincean, Haoyi Qiu, Tim Tjardts, Oleg Lupan, Dumitru Untilă, Cenk Aktas, Rainer Adelung, Liviu Leontie, Aurelian Carlescu, Silviu Gurlui, Mihail Caraman

**Affiliations:** 1 Faculty of Physics and Engineering, Moldova State University, 60 Alexei Mateevici Str., MD-2009 Chisinau, Moldova; veaceslav.sprincean@usm.md (V.S.); dumitru.untila@yahoo.com (D.U.); mihail.caraman@usm.md (M.C.); 2 Functional Nanomaterials, Faculty of Engineering, Institute for Materials Science, Kiel University, Kaiserstr. 2, D-24143 Kiel, Germany; haq@tf.uni-kiel.de (H.Q.); ra@tf.uni-kiel.de (R.A.); 3 Multicomponent Materials, Institute for Materials Science, Kiel University, Kaiserstr. 2, D-24143 Kiel, Germany; tt@tf.uni-kiel.de (T.T.); oca@tf.uni-kiel.de (C.A.); 4 Center for Nanotechnology and Nanosensors, Department of Microelectronics and Biomedical Engineering, Technical University of Moldova, 168, Stefan cel Mare Av., MD-2004 Chisinau, Moldova; 5 Department of Physics, University of Central Florida, Orlando, FL 32816-2385, USA; 6 Faculty of Physics, Alexandru Ioan Cuza University of Iasi, 11 Carol I, 700506 Iasi, Romania; lleontie@uaic.ro (L.L.); sgurlui@uaic.ro (S.G.); 7 Science Research Department, Institute of Interdisciplinary Research, Research Center in Environmental Sciences for the North-Eastern Romanian Region (CERNESIM), Alexandru Ioan Cuza University of Iasi, 11 Carol I, 700506 Iasi, Romania

**Keywords:** chalcogenides, gallium(III) trioxide, native oxide, Eu doping, single crystals, layers, optical properties, photoluminescence

## Abstract

This work studies the technological preparation conditions, morphology, structural characteristics and elemental composition, and optical and photoluminescent properties of GaSe single crystals and Eu-doped *β*–Ga_2_O_3_ nanoformations on *ε*–GaSe:Eu single crystal substrate, obtained by heat treatment at 750–900 °C, with a duration from 30 min to 12 h, in water vapor-enriched atmosphere, of GaSe plates doped with 0.02–3.00 at. % Eu. The defects on the (0001) surface of GaSe:Eu plates serve as nucleation centers of *β*–Ga_2_O_3_:Eu crystallites. For 0.02 at. % Eu doping, the fundamental absorption edge of GaSe:Eu crystals at room temperature is formed by *n* = 1 direct excitons, while at 3.00 at. % doping, Eu completely shields the electron–hole bonds. The band gap of nanostructured *β*–Ga_2_O_3_:Eu layer, determined from diffuse reflectance spectra, depends on the dopant concentration and ranges from 4.64 eV to 4.87 eV, for 3.00 and 0.05 at. % doping, respectively. At 0.02 at. % doping level, the PL spectrum of *ε*–GaSe:Eu single crystals consists of the *n* = 1 exciton band, together with the impurity band with a maximum intensity at 800 nm. Fabry–Perrot cavities with a width of 9.3 μm are formed in these single crystals, which determine the interference structure of the impurity PL band. At 1.00–3.00 at. % Eu concentrations, the PL spectra of GaSe:Eu single crystals and *β*–Ga_2_O_3_:Eu nanowire/nanolamellae layers are determined by electronic transitions of Eu^2+^ and Eu^3+^ ions.

## 1. Introduction

Gallium monoselenide (GaSe) is one of the outstanding representatives of group III–VI lamellar, quasi-two-dimensional (2D) materials, with a direct band gap of 2.00 eV and pronounced anisotropy of mechanical, electrical and optical properties [1,2,3].

Its single crystals exhibit a typical layered structure, each layer being composed of elementary stratified Se–Ga–Ga–Se (Chalcogen–Metal–Metal–Chalcogen) packages, with predominantly covalent bonding inside a package and weak polarizational bonds, of the van der Waals type, between the packages [4,5,6]. Depending on the mutual arrangement of elementary planar packages along the C6 axis, several polytypes are distinguished: *ε*, *β* and *δ*, displaying hexagonal lattice, and *γ*, with rhombohedral crystal lattice. In crystals obtained by the Bridgman–Stockbarger technique, *ε* polytype is reported to be predominant [1,4]. The weak bonds between planar packages facilitate obtaining 2D ultrathin lamellae, which exhibit special optoelectronic properties [7,8]. Based on GaSe nanolayers, a broadband photodetector with a photoresponse of 4.5 A/W at ~120 °C was fabricated [8].

As demonstrated in works [9,10,11,12], doping with isovalent elements (Al, In, Er and Tm) of *ε*–GaSe crystals leads to significant deformation of their hexagonal crystal lattice and also influences their optical and luminescence properties. Aluminum, in small amounts (*c* ≈ 0.01–0.05 at. %), is able to liquidate the vacancies in the gallium sublattice, thus contributing to the increase of the absorption coefficient in the center of *n* = 1 exciton band; whilst, at higher concentrations (*c* ≥ 0.2 at. %), the defects induced by dopant shield the exciton bonds, which is manifested by the decrease in the intensity of the exciton absorption band. Indium as a dopant engenders structural defects in *ε*–GaSe which, together with the shielding of electron–hole bonds, lead to the formation of donor–acceptor pairs and of the impurity PL band [10]. Also, the rare earth elements (Er and Tm) are able to occupy gallium vacancies [11,12]. By doping GaSe with Tm^3+^, luminescence centers are formed in the near-IR region, while Er, in low concentrations, can form localized states within the GaSe bandgap, responsible for its red PL.

The surface of GaSe plates, kept for a long time in a normal atmosphere, is covered with a nanosized gallium oxide layer [13], while as a result of the heat treatment in an atmosphere enriched with oxygen and water vapor, a layer of *β*–Ga_2_O_3_ nanoformations is formed on the surface of GaSe plates [14,15,16]. Under the presence of water vapor and ultraviolet (UV) radiation, the formation of Ga_2_O_3_ and SeO_2_ oxides on thin GaSe plates’ surface is stimulated [16,17]. Since the valence bonds are practically closed at the (0001) surface of GaSe plates, the formation process of *β*–Ga_2_O_3_ oxide is initiated on the edge or in high surface defect density regions of GaSe plates.

In [18], Eu-doped *β*–Ga_2_O_3_ nanowires were obtained by consecutive heat treatments, at temperatures of 1500 °C and 1350 °C, of nanowires obtained from the vapor phase. As a characteristic feature, the cathodoluminescence spectrum of *β*–Ga_2_O_3_:Eu^3+^ nanowires contains the Eu^3+^ emission band with a maximum intensity at 610 nm. In the works [19,20], Chen and co-authors obtained, appealing to the PLD technique and using a mixture of *β*–Ga_2_O_3_ and Eu as evaporation material, *β*–Ga_2_O_3_:Eu thin films exhibiting intense red and violet luminescence, determined by radiative transitions of Eu^3+^ and Eu^2+^ ions, respectively.

The *β*–Ga_2_O_3_ is an *n*–type semiconductor with an ultra-wide energy band gap (4.80–4.90 eV) and moderate concentration of majority charge carriers [21,22]. Since undoped GaSe is a *p*–type semiconductor, through heat treatment in a water vapor-rich atmosphere (AVH_2_O), nanoscale n/p *β*–Ga_2_O_3_/GaSe heterostructures can be obtained.

This paper studies the surface photoluminescence (PL) of the GaSe plates doped with Eu, and the composition and optical properties of the *β*–Ga_2_O_3_:Eu^3+^ layer formed on the surface of GaSe:Eu plates following the heat treatment in an AVH_2_O at temperatures below the melting point.

## 2. Materials and Methods

Undoped and Eu-doped GaSe single crystals with doping concentrations of 0.02, 0.05, 0.50, 1.00 and 3.00 at. % were grown using the Bridgman–Stockbarger technique [23]. Ga (5N) and Se (5N), in stoichiometric proportions, were used as primary chemical elements, while Eu (5N), as a dopant. The synthesis and growth of single crystal samples were carried out in one cycle, in a three-zone furnace. Quartz ampoules with an internal diameter of 14–15 mm were used. To avoid material contamination, the internal surface of ampoules was covered with a graphite layer, obtained through high-temperature acetone pyrolysis. In this way, single crystals with a diameter of 14–15 mm and a mass of ~20 g were obtained. By mechanical splitting in a direction perpendicular to the C6 axis, from undoped GaSe single crystals and those doped with Eu, in concentrations lower than 0.05 at. %, plates with thicknesses in submicrometer to millimeter range, smooth surfaces and without microscopic defects, were obtained. Single crystal GaSe:Eu plates were subjected to heat treatment at temperatures of 750, 850 and 900 °C, with durations from 30 min to 12 h, in humid air. It was observed that following heat treatments at 850 and 900 °C, the surfaces of GaSe:Eu plates are covered with a white oxide layer, which strongly diffuses white light. As a result of the heat treatment at 750 °C, it is observed that the white oxide layer is formed only on the edges of GaSe:Eu plates.

The crystal structure and surface morphology of heat-treated samples were studied via X-ray diffraction (XRD), using a Sefert 300TT diffractometer (wavelength *λ_CuKα_* = 1.54060 Å, 40 kV, 40 μA) and scanning electron microscopy (SEM) (Remzeitss type apparatus at 7 kV and of 8 mA), respectively; elemental and structural composition of *β*–Ga_2_O_3_:Eu layer was studied using XPS (X-ray photon spectroscopy) technique, with an Omicron-Nano-Technologic GmbH spectrometer with aluminum anticathode and micro-Raman (Witec alpha300 RA)-integrated Raman/AFM microscope. The calibration of the collected electric charge was carried out according to the C1s carbon line located at 284.80 eV. The XPS spectra were analyzed using the Casa XPS program, version 2.3.23. The photoluminescence of the samples was studied with spectrophotometric equipment based on an MDR-2 type monochromator, with a diffraction of 600 mm^−1^. The energy resolution at 500 nm and 800 nm wavelengths was 2.00 meV and 1.00 meV, respectively. Photoluminescence of *β*–Ga_2_O_3_:Eu nanowire and nanoribbon assemblies was excited with a UV-light band covering the wavelength range between 248 and 260 nm, centered at 253.5 nm, selected from the emission spectrum of a 500 W mercury vapor lamp, using a quartz prism monochromator with a linear dispersion of 3.0 nm/mm at 257.6 nm wavelength. Photoluminescence of undoped GaSe crystals, at 293 K, was excited with a 405 nm (3.00 eV) laser radiation (power density of 150 µW/cm^2^), while PL excitation of Eu-doped GaSe crystals was performed by means of an N_2_ laser [337.4 nm (3.67 eV)], with a power density of ~1.5 × 10^3^ W/cm^2^. The PL intensity was recorded using a photomultiplier with multi-alkali [(Na_2_K)Sb + Cs] photocathode.

## 3. Results and Discussion

**Figure 1:**
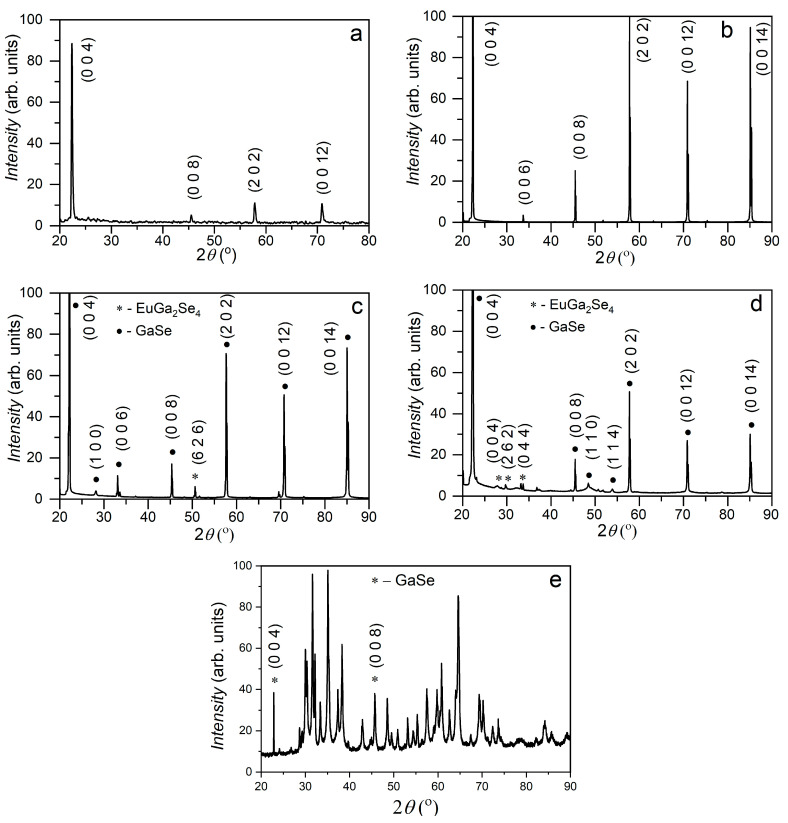
XRD plots of GaSe single crystals, undoped (**a**) and Eu-doped with different concentrations: 0.05 at. % (**b**), 1.00 at. % (**c**), 3.00 at. % (**d**); material obtained through 12 h heat treatment, at 900 °C, in water vapor-enriched atmosphere, of GaSe:Eu (3.00 at. %) plates (**e**).

Figure 1 shows XRD diagrams for undoped (a) and Eu-doped [0.05 at. % (b), 1.00 at. % (c) and 3.00 at. % (d)] GaSe single crystals. In the 2*θ* angular range of 20–90°, reflections from the (002), (004), (008), (202) and (0012) atomic planes are emphasized in recorded diffractograms, which, according to PDF card no. 00-037-0931, correspond to the *ε*–GaSe polytype-hexagonal lattice (space group P63/mmc) with parameters *a* = 3.749 Å, *c* = 15.907 Å and *γ* = 120.00°.

As shown in Figure 1b, in the XRD plot of GaSe crystals doped with Eu in concentrations up to 0.05 at. %, the characteristic reflections of the undoped material are present. In the XRD diagram of GaSe:Eu (1.00 at. %) single crystals (Figure 1c), together with the intense diffraction lines of GaSe (hexagonal lattice), low-intensity reflections are shown at 2*θ* angles of 29.71, 36.83 and 50.62°. As can be seen by comparing the diagrams in Figure 1c,d, the intensity of these lines is increasing together with the increase in Eu concentration from 1.00 to 3.00 at. %. At the same time, in Figure 1d, reflections positioned at 48.00 and 51.81° are clearly emphasized, which according to PDF card no. 01-070-2524, correspond to the EuGa_2_Se_4_ compound with orthorhombic crystal lattice (space group Fddd) with parameters *a* = 21.579 Å, *b* = 21.336 Å, *c* = 12.736 Å and *α* = *β* = *γ* = 90°. Therefore, in materials with Eu concentrations of 0.05, 1.00 and 3.00 at. %, GaSe single crystals with hexagonal lattice also contain EuGa_2_Se_4_ crystallites, with orthorhombic structure.

As a result of heat treatment (at 900 °C, for 12 h) in AVH_2_O, on the surface of GaSe:Eu plates, a white layer is formed, which intensely diffuses the incident light. Figure 1e shows the X-ray diffractogram of the material obtained using a GaSe:Eu (3.00 at. %) plate, ~120 μm thick, as the primary material. For 2*θ* angles ranging from 20 to 90°, two reflections are emphasized, that can be ascribed to (004) and (008) crystal planes of GaSe, hexagonal lattice (PDF card no. 00-037-0931). Several diffraction lines corresponding to *β*–Ga_2_O_3_ (PDF card no. 43-1012), with monoclinic lattice (space group C2/m and lattice parameters *a* = 12.23 Å, *b* = 3.04 Å, *c* = 5.800 Å and *β* = 103.7°) were also identified. At the same time, this diagram exhibits a series of low-intensity lines, located at 26.78, 26.82, 32.20, 53.15 and 55.80°. These reflections are characteristic of europium–gallium garnet (Eu_3_Ga_5_O_12_), formed in the growth process of *β*–Ga_2_O_3_:Eu (3.00 at. %) crystal fibers [24].

**Figure 2:**
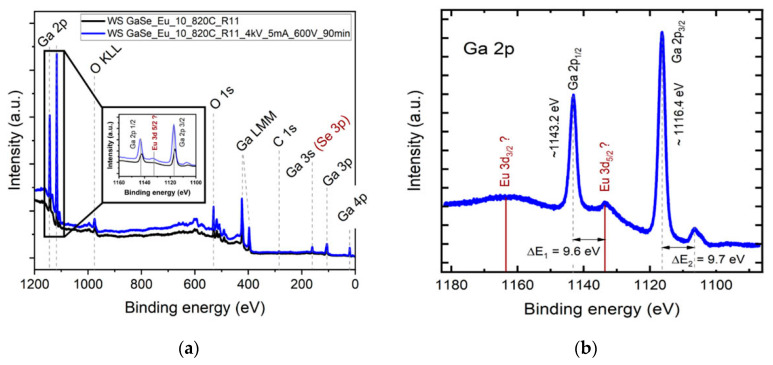
(**a**) XPS survey spectra of the heat-treated GaSe:Eu sample before and after a total of 90 min of Ar cleaning, indicating the presence of O, C and Ga at the sample surface. The inset graph shows the Ga 2p region (higher resolution). A slight increase in the intensity of Eu-related lines can be observed after Ar cleaning. (**b**) High-resolution scan of the Ga 2p signal with Eu 3d as a reference, according to [6]. ΔE1 and ΔE2 shifts, with respect to the main peaks, are emphasized.

The elemental surface composition of the layer obtained via heat treatment of GaSe plates doped with 3.00 at. % Eu, at a temperature of 850 °C, in a water vapor-enriched atmosphere, for 6 h, was studied through XPS spectroscopy. A 300 W Al anticathode tube (*E_Alka_* = 1486.6 eV) was used as an X-ray source (*λ_Alka_* = 8.3402 Å). The XPS spectrum was analyzed with CasaXPS processing software (version 2.3.23). Previously, the sample surface was cleaned with an Ar ion beam (5 mA, 4 kV), at a focusing voltage of 600 V. In Figure 2a, the XPS spectrum of heat-treated GaSe:Eu, at photon energy from 0 to 1200 eV is shown. Within this energy range, the following emission lines were found to be present: galium—Ga 2p, Ga LMM, Ga 3s, Ga 3p, Ga 4p; oxygen—O KLL and O 1s; it is also worth noting that small peaks of Eu 3d3/2 and Eu 3d5/2 emission lines were present. In Figure 2b, the 1100–1180 eV region of the high-resolution spectrum is presented, in which a peak located at 1133.6 eV is well emphasized; according to the studies reported in [25], it could be interpreted as an Eu 3d5/2 signal originating from the Eu^3+^ ion in Eu_2_O_3_.

As can be seen from Figure 2b, at energies corresponding to Eu^3+^ 3d5/2 emission, a poorly outlined peak is emphasized in the high-resolution XPS spectrum. The low peak intensity of this line is probably determined by the low Eu^3+^ concentration in the surface layer of the sample. In [26], the XPS spectrum of Eu^3+^ ions in ZnO nanoparticles is analyzed, from which it can be clearly seen that the Eu^3+^ 3d3/2 signal is ~2 times smaller compared to that of Eu^3+^ 3d5/2. At the same time, the emission lines of carbon are well emphasized in the recorded XPS spectrum, which are probably caused by the sample adsorption of hydrocarbons from the atmosphere.

**Figure 3:**
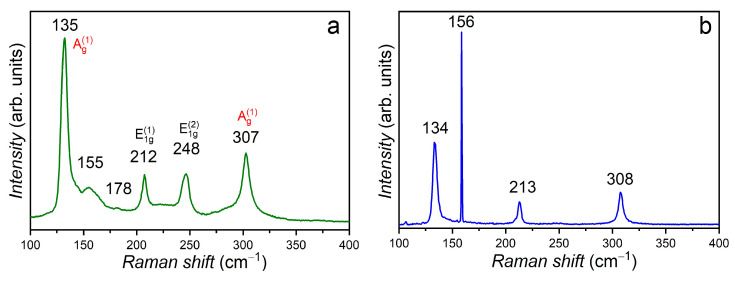

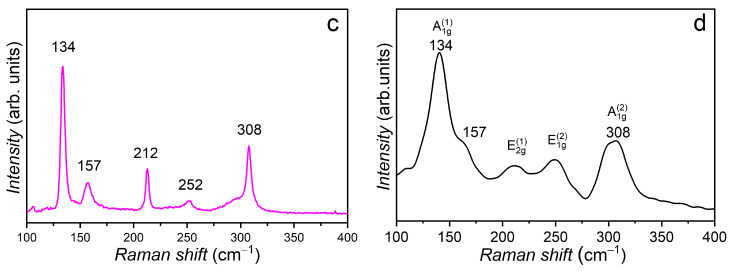
Raman spectra of GaSe:Eu single crystals for doping concentrations: (**a**) 0.02 at. %, (**b**) 0.05 at. %, (**c**) 1.00 at. % and (**d**) 3.00 at. %.

Valuable complementary information on the surface composition of GaSe:Eu plates, for doping concentrations between 0.02 and 3.00 at. %, can be obtained from the analysis of Raman spectra (Figure 3). The *ε*–GaSe polytype is a typical representative of the point group D3h, displaying 12 vibration modes. The planar vibrations *E*′ and *E*″ and non-planar vibrations *A_*1*_*′ and *A_*2*_*″ are known to be active in Raman spectra [26,27].

**Figure 4:**
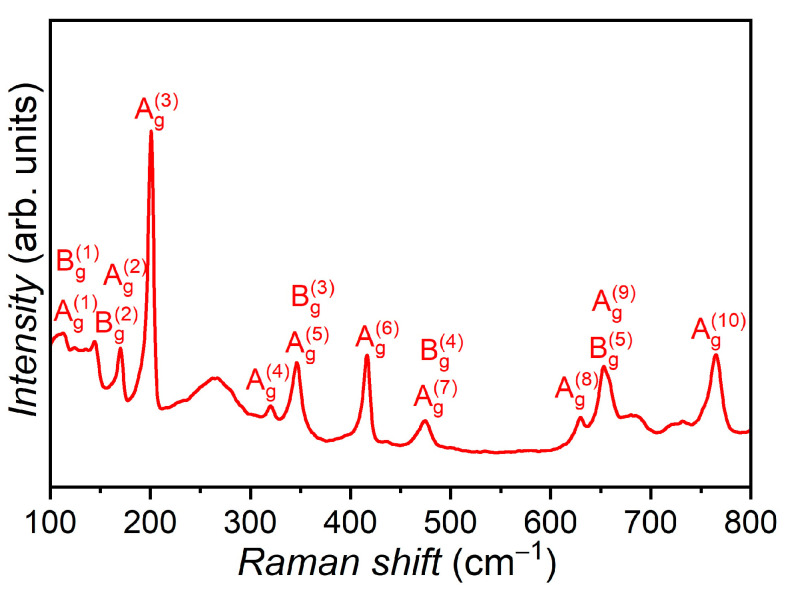
Raman spectra of GaSe:Eu (3.00 at. %) after annealing in a humid atmosphere at 900 °C for 6 h.

At 480 nm excitation wavelength of Raman spectra, the absorption coefficient (*α*) in GaSe crystals is equal to ~104 cm^−1^ (Figure 4). Therefore, one can assume that the thickness of the excited GaSe layer in Raman spectra does not exceed ~2 μm.

Figure 3a shows the Raman spectrum of the GaSe plate doped with 0.02 at. % Eu, in which four intense peaks located at 135 cm^−1^ [A_1g_^(1)^], 212 cm^−1^ [E_2g_^(1)^], 248 cm^−1^ [E_1g_^(2)^] and 307 cm^−1^ [A_1g_^(2)^] are present; these are consistent with the Raman vibration modes in hexagonal GaSe single crystals, as reported in [7,28,29]. In the Raman spectrum of GaSe:Eu (0.02 at. %) sample (Figure 3a), together with the characteristic vibration peaks of GaSe (hexagonal lattice) with the symmetries A_1g_^(1)^, E_2g_^(1)^, E_1g_^(2)^ and A_1g_^(2)^, a low-intensity peak with a broad contour is also present, with maximum peak located at 178 cm^−1^. This peak has been also observed in the Raman spectra of Eu_2_O_3_ [30]. The peak positioned at 155 cm^−1^ correlates well with the LO vibration mode in EuSe [31]. It is likely that oxygen could be introduced in the synthesis process of the compound together with metallic Eu, since the Eu(OH)_3_ hydroxide can be formed at room temperature in the atmosphere [32]. At high temperatures, this hydroxide decomposes into Eu_2_O_3_ and water [$2Eu{\left(OH\right)}_{3}\overset{300–400\;{}^{\circ}C}{\to }{Eu}_{2}O_{3}+3H_{2}O$].

The Raman spectra of GaSe plates doped with 0.05, 1.00 and 3.00 at. % Eu display a similar structure (Figure 3b–d).

The intense peaks in these spectra correspond to the lattice vibrations of *ε*–GaSe (hexagonal structure), and the presence of Eu dopant is manifested by two low-intensity peaks, positioned at 119 and 155 cm^−1^, which can be associated with the lattice vibrations of Eu_2_O_3_ and cubic EuSe [31,32,33]. In the wavenumber ranging between 100 and 800 cm^−1^, the Raman spectrum of single crystal GaSe plate doped with 3.00 at. % Eu, subjected to heat treatment in a humid atmosphere at 900 °C for 6 h, contains 11 peaks located at 114, 132, 148, 201, 318, 348, 416, 468, 628, 654 and 764 cm^−1^ (Figure 4). The mentioned structure of the Raman spectrum can be attributed to the monoclinic *β*–Ga_2_O_3_ lattice [34,35,36,37].

The oxidation process of *ε*–GaSe plates at temperatures below the melting point, in the presence of oxygen or in an oxygen and water vapor-enriched inert gas atmosphere can be described by the reactions [9,10,17]:

$$\left[6GaSe+3O_{2}\to {Ga}_{2}{Se}_{3}+{2Ga}_{2}O_{3}+3Se\right]\;{[Ga}_{2}{Se}_{3}+3/2O_{2}\to {Ga}_{2}O_{3}+3Se]\;\left[2GaSe+3H_{2}O+5/2O_{2}\to {Ga}_{2}O_{3}+H_{2}SeO_{3}+2H_{2}O\right]\;[H_{2}SeO_{3}\to H_{2}O+Se+O_{2}]$$

**Figure 5:**
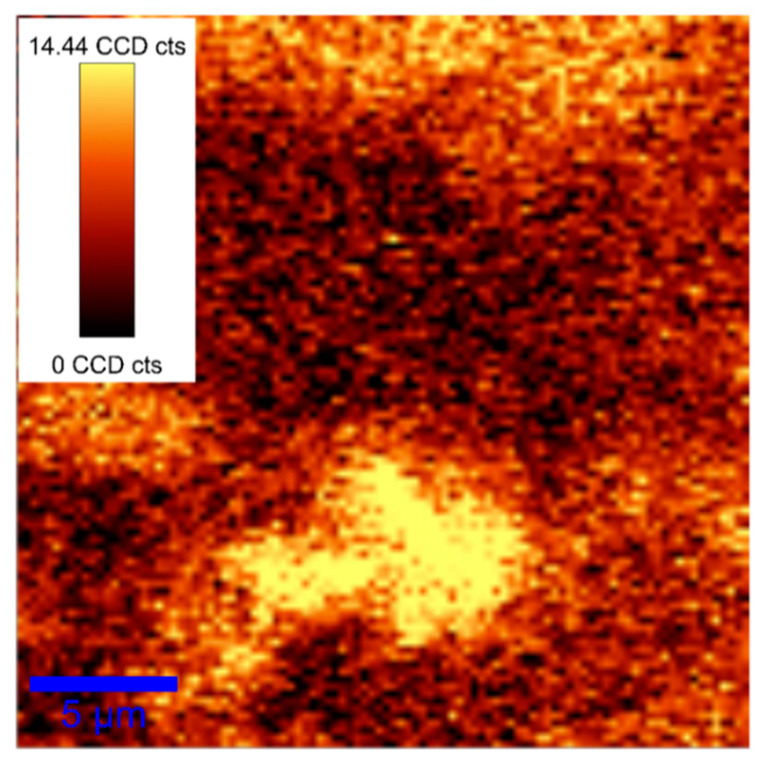
Filtered Raman image of GaSe:Eu (3.00 at. %) after annealing at 900 °C for 6 h, in terms of mode $A_{g}^{\left(3\right)}$ (201 cm^−1^) of Ga_2_O_3_.

As can be seen from the microscopy image (Figure 5), as a result of heat treatment in a humid atmosphere, at 900 °C for 6 h, an island layer of *β*–Ga_2_O_3_ is formed on the surface of GaSe:Eu (3.00 at. %) plate, in accordance to the fact that the surface defects of GaSe:Eu plate can serve as growth centers of *β*–Ga_2_O_3_ crystallites.

**Figure 6:**
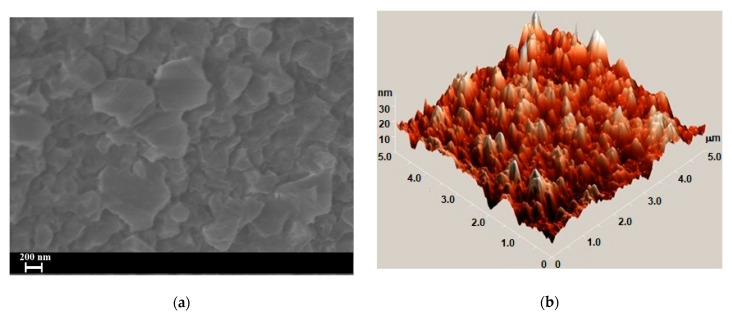
(**a**) SEM image of *β*–Ga_2_O_3_:Eu^3+^ layer formed on the surface of single crystalline GaSe plate doped with 1.00 at. % Eu; (**b**) AFM image of an island sector of *β*–Ga_2_O_3_:Eu^3+^ layer.

The micrometer-island structure of *β*–Ga_2_O_3_:Eu layer is well emphasized in the SEM micrograph of the GaSe plate, doped with 1.00 at. % Eu, subjected to heat treatment in a humid atmosphere, at 850 °C, for 6 h (Figure 6a). As can be seen from the AFM image (Figure 6b), *β*–Ga_2_O_3_:Eu formations are tower- and cone-shaped and exhibit submicrometric bases.

The fundamental absorption edge of *ε*–GaSe single crystals is well studied and is determined by the excitonic absorption mechanism [38,39,40]. The absorption coefficient (*α*) of radiation with wavelength *λ*, in both specially undoped and 3.00 at. % Eu-doped *ε*–GaSe single crystals, was determined from measurements of the transmittance (t) and Fresnel reflection coefficient (R) of the sample surface using the relation [41]:

T = (1 − R)^2^ exp(−*α*d)/[1 − R^2^ exp(−2*α*d)], (1)

where d is the thickness of the plane-parallel sample.

Since the layer formed on the surface of Eu-doped GaSe plates after heat treatment at 900 °C (*β*–Ga_2_O_3_:Eu) is microgranular and strongly diffuses the incident light, the absorption in the vicinity of the fundamental absorption edge of this layer was determined from measurements of the diffuse reflectance (R_d_), using the Kubelka–Munk function [42]:

F(R_d_)·(1 − R_d_)^2^/(2R_d_) = *α*/S (2)

where *α* is the absorption coefficient, and S is the radiation scattering factor (diffusion coefficient), which for grain sizes greater than the incident light wavelength is a constant quantity. Therefore, one can write:

*α* = F(R_d_)·S (3)

**Figure 7:**
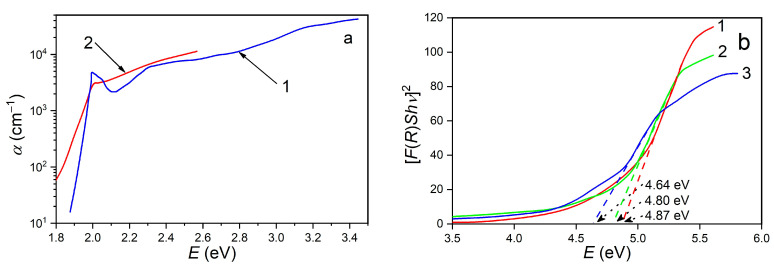
(**a**) Absorption spectra of GaSe:Eu crystals for doping concentrations: 1—0.02 at. % and 2—3.00 at. %. (**b**) Determining fundamental absorption edge of nanostructured *β*–Ga_2_O_3_:Eu layer on GaSe:Eu substrate for doping concentrations: 1—0.02 at. %; 2—0.50 at. % and 3—3.00 at. %.

In Figure 7a, absorption spectra of GaSe crystals doped with 0.02 and 3.00 at. % Eu are presented. In the region of the fundamental absorption edge of GaSe:Eu (0.02 at %) crystals, the absorption peak with the formation of *n* = 1 exciton, located at 1.999 eV (photon energy), is well emphasized. The direct band gap of GaSe crystals, at 293 K, is equal to 2.020 eV [43].

The binding energy of the electron–hole pair in undoped GaSe crystals is 20.6 meV [38], indicating that at room temperature, the excitons are partially ionized. As can be seen from Figure 7a, curve 2, Eu, as a dopant with a concentration of 3.00 at. %, is able to effectively shield the exciton bonds in GaSe:Eu. At the same time, from curve 1, it can be seen that in the photon energy range from 2.30 eV to 3.50 eV, the absorption coefficient in the depth of the fundamental absorption band of GaSe doped with a low Eu concentration (0.02 at. %) increases slowly together with photon energy, from 6 × 10^3^ to 4 × 10^4^ cm^−1^. The comparison with the results of previous research reported in [40] demonstrates that the spectral distribution of the absorption coefficient in the depth of the fundamental absorption band of GaSe single crystals weakly depends on the presence of Eu, for moderate concentrations.

In [44], the influence of Al impurities on the absorption spectrum in the region of the fundamental absorption band of GaSe single crystals is studied, from which the absence of excitonic peak by 2.00 eV for 1.00 at. % Al is revealed. The shielding effect of exciton (electron–hole) bonds in *ε*–GaSe crystals is enhanced in the case of rare-earth doping. The exciton bonds are completely shielded at a doping level of 0.098 at. % Eu in GaSe crystals [45].

As can be seen from the SEM images of *β*–Ga_2_O_3_:Eu layers on GaSe:Eu substrate (Figure 6), the grains producing the light diffusion exhibit submicrometer sizes. Therefore, in the wavelength region *λ* ≤ 300 μm, one can consider in formula (2) that *α*~F(R) and, consequently, the direct band gap of *β*–Ga_2_O_3_:Eu layer can be determined by extrapolating the Kubelka–Munk function to zero (Figure 7b), using the relation [46]:

[F(R)hν]^2^ = A(hν − E_g_). (4)

Here, A denotes a constant that is independent of photon energy. As shown in Figure 7b, the direct band gap decreases from 4.87 eV, for *β*–Ga_2_O_3_ on GaSe:Eu (0.05 at. %) to 4.64 eV for *β*–Ga_2_O_3_:Eu on GaSe:Eu (3.00 at. %). The magnitude of the energy band gap of micro- and nanostructured *β*–Ga_2_O_3_ layers depends on many factors, such as manufacturing technology, dopant type and concentration, and substrate nature [47,48,49,50].

The photoluminescence of undoped and Eu-doped (0.02, 0.05, 0.50, 1.00 and 3.00 at. %) GaSe single crystals was excited with laser radiation (wavelength *λ* = 405 nm and power density *P* = 5 mW/cm^2^) at room temperature (293 K). The thickness of the excited layer, of ~2 μm, was estimated by taking into account the value of the absorption coefficient at this wavelength, which was equal to ~1 × 10^4^ cm^−1^ (Figure 7a) (within this layer thickness, the radiation intensity is attenuated by ~9 times).

**Figure 8:**
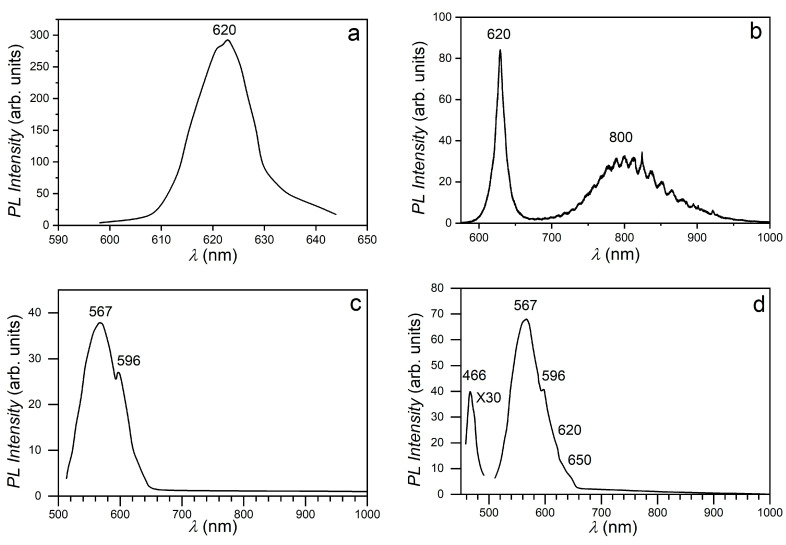

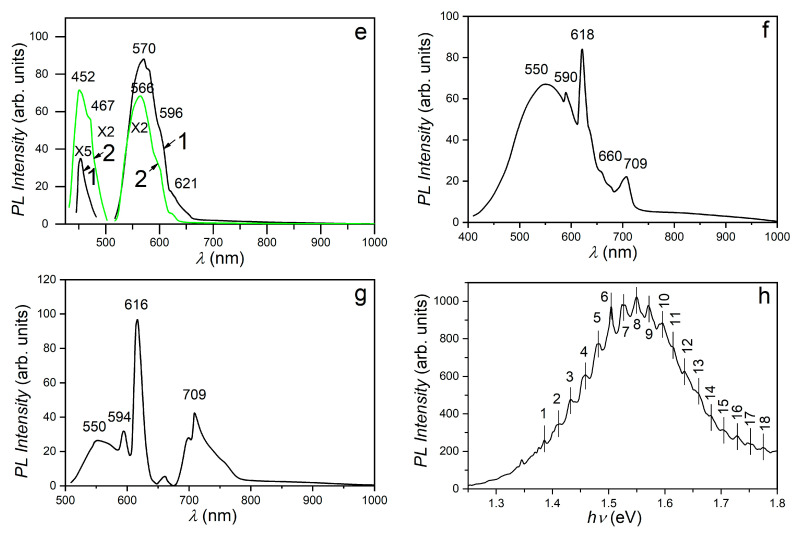
PL spectra at room temperature upon *λ_ex_* = 405 nm wavelength (45 μW/cm^2^) excitation: (**a**) undoped GaSe; (**b**) GaSe:Eu (0.025 at. %); (**c**) GaSe:Eu (0.05 at. %); (**d**) GaSe:Eu (0.50 at. %); (**e**) GaSe:Eu (1.00 at. %) (curve 1) and the *β*–GaSe:Eu (3.00 at. %) (curve 2). Material obtained through heat treatment in the humid atmosphere of *ε*–GaSe:Eu (3.00 at. %) at 900 °C for 6 h (**f**) and 12 h (**g**), upon *λ_ex_* = 254 nm (**f**) and *λ_ex_* = 405 nm (**g**) excitation. Contour of the red PL band of GaSe:Eu (0.02 at. %) crystals at 23 °C and *λ_ex_* = 405 nm (1.2 mW/cm^2^) (**h**).

Figure 8a shows the PL spectrum of the single crystalline GaSe plate, which reveals that upon 405 nm excitation, a band with a narrow contoured and maximum peak located at 620 nm (2.00 eV) is formed.

This PL band is probably formed by merging the *n* = 1 exciton emission band (Figure 7a) with the recombination PL band of nonequilibrium charge carriers in the conduction band with holes in the valence band. An analogous spectrum with a maximum of 2.00 eV was obtained in the PL spectrum of GaSe nanosheets grown from the vapor phase on a Si substrate [51,52].

The PL spectrum of GaSe:Eu crystals (0.02 at. %) (Figure 8) is composed of two bands: one with a narrow contour, of excitonic nature, with maximum photon energy of 2.00 eV, and another with a broad contour and oscillatory structure, with maximum photon energy at ~1.54 eV. Probably by doping the sample with 0.02 at. % Eu, in GaSe crystals, ionized centers are formed to which the excitons can be bound, forming complexes with a binding energy of ~10 meV. The presence of the intense room temperature PL band, with a narrow contoured and maximum peak at 620 nm (2.00 eV), is indicative of the high perfection of GaSe single crystals doped with 0.025 at. % Eu. One can admit that Eu, in concentrations of 0.025 at. %, is able to occupy gallium vacancies (V_Ga_). Since the ionic radius of Eu^3+^ (0.96 Å) [53] is greater than that of the Ga^3+^ (0.62 Å), in the V_Ga_ region the deformation of the crystal lattice occurs, with a disturbance of Ga and Se positions.

According to its shape, the PL band with maximum intensity at 1.54 eV is of donor–acceptor type.

The low electrical conductivity of GaSe crystals grown via the Bridgman–Stockbarger technique is determined by the fact that they are strongly compensated [54]. The surface donor level in this material is located at ~12 meV with respect to the *n* = 1 excitonic level [55,56], which contributes to the excessive exciton peak broadening at room temperature (Figure 7a). Taking into account that the exciton binding energy is 20.60 meV, in order to interpret the PL band with maximum intensity at 1.54 eV, it is necessary to admit that at low concentrations, Eu engenders a deep acceptor level in the band gap of GaSe:Eu crystals, located at ~0.447 eV above the top of the valence band. Furthermore, deep acceptor levels can be also formed in the band gap of GaSe single crystals by lanthanides such as Er, Gd [57,58] and isovalent elements In and Al [59].

The parallelism and surface perfection of GaSe plates are necessary requirements for the formation of Fabry–Perrot (FP) cavities, in which interference of photoluminescent radiation may occur. In [52], the PL interference effect was demonstrated in elementary layer assemblies of gallium selenide. Besides, the PL interference effect was observed in FP microcavities in Mn-doped ZnSe nanofibers [10,60]. As can be seen from Figure 8b,h, in the case of near-infrared (NIR) PL band of single crystalline GaSe:Eu (0.02 at %) plates, the interference fringes covering the wavelength ranging from 690 nm up to ~950 nm are well emphasized. The size of the FP cavities can be determined from measurements of the wavelengths *λ_*1*_* and *λ_*2*_* for two consecutive maxima/minima (Figure 8h) and of the average refractive index (*n*) over this wavelength range, using the equation [41]:

l = (1/2*n*)[(*λ*_1_·*λ*_2_)/(*λ*_1_ − *λ*_2_)]. (5)

For a refractive index of GaSe crystals, in the spectral range between 780 and 850 nm, equal to 2.83 [61] and the wavelengths of the interference maxima (minima) in this range, from the above formula, a distance of 9.3 μm is obtained between the reflecting surfaces of the FP cavity.

The fringe contrast factor,

*γ* = l_max_/l_min_, (6)

in the center of the PL band, is equal to 1.25. If we neglect the absorbance inside the FP cavity, then the contrast factor depends only on the reflection coefficient (r) of the cavity surface and is given by the expression [62]:

*γ* = [(1 + *r*)/(1 − *r*)]^2^. (7)

The reflection coefficient, within the frame of this model, is given by:

*r* = [(*n*_1_/*n*_2_ − 1)/(*n*_1_/*n*_2_ + 1)]^2^. (8)

For *γ* = 1.25, from (7) and (8), *n*_1_⁄*n*_2_ =1.64 is obtained. The refractive index of GaSe crystals, at the wavelength *λ* ≈ 800 nm, is *n_⊥_* = 2.86. Therefore, the refractive index of the medium in the FP cavity is equal to 1.74.

When increasing the Eu concentration in the doped GaSe crystals to 0.05 at. %, the PL spectrum transforms into a broad band with a maximum intensity at 567 nm (2.187 eV), located in the region of the fundamental absorption edge of GaSe crystals (Figure 8c), with a threshold at 621 nm (1.998 eV) and a small peak at 596 nm (2.080 eV). Increasing the Eu concentration up to 0.50 at. % leads to an increase in the PL intensity of the 567 nm band by ~5 times; besides, a low-intensity PL band appears in the blue region, with a maximum intensity at 466 nm (2.61 eV) (Figure 8d); when increasing the Eu concentration in GaSe to 1.00 at. % and further, up to 3.00 at. %, the intensity of the yellow band increases, maintaining the peculiarities at 596 nm and 621 nm (Figure 8e). At the same time, the intensity of the blue band increases and its maximum intensity is found to blueshift, located at 452 nm (Figure 8e,f).

The luminescence inside the absorption band of GaSe crystals can be explained if Eu can be found in Eu^1+^ and Eu^2+^ at doping concentrations in the range of 0.05–3.00 at. %. The energies of Eu^+^ ion levels lay in the range of 2.95–3.38 eV [63]. The electron transitions of this ion may correspond to the PL band with a maximum intensity at 452/467 nm. The green-yellow band can be attributed to the electron transitions of the Eu^2+^. Depending on the crystal lattice in which the Eu^2+^ ion is found, emission bands with maxima at wavelengths of 555–565 nm can be obtained [64,65,66].

In the PL spectrum of the GaSe:Eu plates subjected to heat treatment in humid air, at 900 °C, for 6–12 h, through which nanostructured *β*–Ga_2_O_3_:Eu^3+^ layers are formed, new peculiarities appear. As seen from Figure 8f,g, upon excitation with radiation corresponding to the fundamental absorption edge of *β*–Ga_2_O_3_ (*λ_ex_* = 260 nm), the PL spectrum contains, in addition to the PL band with a broad contoured and maximum peak at 550 nm (2.254 eV), two narrow-contour bands with maxima at 590 nm (2.101 eV) and 618 nm (2.006 eV), a plateau by 660 nm (1.878 eV), and a low-intensity broad band with maximum intensity at 709 nm (1.749 eV).

The band with a maximum intensity at 550 nm can be associated with the emission of *β*–Ga_2_O_3_ micro- and nanocrystallites, while the set of bands in the wavelength range from 590 to 750 nm can be interpreted as radiative transitions of Eu^3+^ ion. The structure of the emission spectrum of Eu^3+^ ion in *β*–Ga_2_O_3_ is well revealed in the PL spectrum excited with *λ* = 405 nm (3.06 eV) radiation (Figure 8g). According to the energy level diagram of Eu^3+^ [64], the PL bands with maxima at wavelengths of 590, 616, 660 and 709 nm are determined by electron transitions from the excited Eu^3+^ state, *^*5*^D_*0*_*, to the ground state, *^*7*^F_j_*, with *j* = 1–4. Previously, in the works [20,67,68], fibers and thin films of *β*–Ga_2_O_3_ doped with Eu were prepared, in which the emission spectrum of Eu^3+^ ions is in good agreement with the PL spectra of actual *β*–Ga_2_O_3_:Eu nanoformations obtained through heat treatment, in humid atmosphere, of GaSe single crystals doped with 3.00 at. % Eu, shown in Figure 8g.

## 4. Conclusions

Through the Bridgman–Stockbarger technique, single crystals of GaSe doped with 0.02, 0.05, 0.50, 1.00 and 3.00 at. % Eu were obtained.

The fundamental absorption edge in crystals with low Eu concentration (*c* ≤ 0.025 at. %) is formed by excitons in the *n* = 1 state.

Europium, in small amounts (*c* ≈ 0.025 at. %), together with the liquidation of Ga vacancies in the gallium sublattice, forms micrometer-sized resonant cavities in single crystalline GaSe plates, filled with an IR transparent material with a refractive index of ~1.74.

Europium as a dopant, in amounts greater than 0.05 at. %, effectively shields the exciton bonds. For these concentrations, Eu is able to form EuSe and Eu_2_O_3_ crystallites in hexagonal GaSe crystals.

Heat treatment of GaSe:Eu plates at 900 °C for 12 h, in a humid atmosphere, leads to the formation of a nanostructured *β*–Ga_2_O_3_ layer doped with Eu on the plates’ surface.

At doping concentrations from 0.05 at. % to 3.00 at. %, Eu can be found in Eu^+^ and Eu^2+^ states, while in the nanostructured *β*–Ga_2_O_3_ layer formed on GaSe:Eu (3.00 at. %) substrate, Eu^3+^ ionized state is active.

The direct band gap in *β*–Ga_2_O_3_:Eu layers depends on the dopant (Eu) concentration and is decreasing from 4.87 eV, for an Eu concentration of 0.05 at. %, up to 4.64 eV, at 3.00 at. % concentration.

## Data Availability

Data are contained within the article.
